# Identification of miRNAs and Their Targets in Cotton Inoculated with *Verticillium dahliae* by High-Throughput Sequencing and Degradome Analysis

**DOI:** 10.3390/ijms160714749

**Published:** 2015-06-30

**Authors:** Yujuan Zhang, Wei Wang, Jie Chen, Jubo Liu, Minxuan Xia, Fafu Shen

**Affiliations:** State Key Laboratory of Crop Biology, College of Agronomy, Shandong Agricultural University, Tai’an 271018, China; E-Mails: xiaoyunv_1124@126.com (Y.Z.); sdauww@126.com (W.W.); chenj_19@163.com (J.C.); liujubo2014@163.com (J.L.); Xiaj_19@163.com (M.X.)

**Keywords:** cotton, *Verticillium dahliae* Kleb., microRNA, high-throughput sequencing, degradome

## Abstract

MicroRNAs (miRNAs) are a group of endogenous small non-coding RNAs that play important roles in plant growth, development, and stress response processes. Verticillium wilt is a vascular disease in plants mainly caused by *Verticillium dahliae* Kleb., the soil-borne fungal pathogen. However, the role of miRNAs in the regulation of Verticillium defense responses is mostly unknown. This study aimed to identify new miRNAs and their potential targets that are involved in the regulation of Verticillium defense responses. Four small RNA libraries and two degradome libraries from mock-infected and infected roots of cotton (both *Gossypium* *hirsutum* L. and *Gossypium* *barbadense* L.) were constructed for deep sequencing. A total of 140 known miRNAs and 58 novel miRNAs were identified. Among the identified miRNAs, many were differentially expressed between libraries. Degradome analysis showed that a total of 83 and 24 genes were the targets of 31 known and 14 novel miRNA families, respectively. Gene Ontology analysis indicated that many of the identified miRNA targets may function in controlling root development and the regulation of Verticillium defense responses in cotton. Our findings provide an overview of potential miRNAs involved in the regulation of Verticillium defense responses in cotton and the interactions between miRNAs and their corresponding targets. The profiling of these miRNAs lays the foundation for further understanding of the function of small RNAs in regulating plant response to fungal infection and Verticillium wilt in particular.

## 1. Introduction

MicroRNAs (miRNAs) are a class of endogenous non-coding small RNAs (sRNAs) that regulate gene expression at the transcriptional and post-transcriptional levels via mRNA cleavage or translational repression in plants and animals [1,2,3]. In higher plants, miRNAs play important roles in growth, development, stress responses, and many other biological processes [4,5,6]. In particular, there is increasing evidence that miRNAs are involved in regulating abiotic and biotic stress responses, including disease resistance [7,8,9,10]. Recently, sRNA-mediated gene silencing was found to play a significant role in plant defense against pathogens [8,11,12,13]. In *Arabidopsis thaliana*, miR393 was found to contribute to basal defense against *Pseudomonas syringae* by regulating auxin signaling [8]. miR393 can be induced upon perception of flg22 (a 22-amino acid peptide), a PAMP (pathogen-associated molecular pattern) derived from bacterial flagellin, and negatively regulates transcripts of a number of F-box auxin receptors [8]. Moreover, miR160, miR167, and miR393 were identified as highly induced after infection using sRNA expression profiling on *Arabidopsis* leaves collected at 1 and 3 h post-inoculation of *Pseudomonas syringae pv.* tomato (DC3000hrcC). [12]. Interestingly, all three miRNAs negatively regulate auxin signaling by either targeting auxin receptor genes or auxin response factors [12]. Another recent study reported that miR162 and miR168 targeted Dicer-like1 and Argonaute proteins, which are likely up-regulated by infection and presumably positively regulated by plant defense responses, although their functions need to be confirmed by experimental data [13].

Cotton is one of the most important economic crops in the world. It is highly susceptible to cotton Verticillium wilt, a disease that significantly affects cotton yield and quality. Verticillium wilt is the primary disease attacking cotton crops and is mainly caused by a soil-borne fungal pathogen, *Verticillium dahliae* Kleb. (*V. dahliae*) [14]. The representative symptoms of susceptible cotton include leaf curl, necrosis and defoliation, and stem wilt [15]. It leads to discoloration of cotton leaves and stem vascular bundles, and it inhibits photosynthesis and increases respiration [16]. However, there is no proven control (chemical or cultural) for this disease as the mechanisms of Verticillium wilt remain poorly understood. Despite great efforts in producing wilt-resistant cotton cultivars by traditional breeding, very few *Gossypium hirsutum* varieties—the main species of cotton currently cultivated in the world—are resistant to the wilt [17]. Therefore, identification of new miRNAs and elucidation of their functions in response to *V. dahliae* infection will help us understand the regulation of pathogen defense responses. Recently, the *Gossypium raimondii* genome sequence was completed [18,19], and this will greatly advance biological research on cotton.

Although many cotton miRNAs were identified in previous research, the role of miRNAs in the regulation of Verticillium defense responses is mostly unknown. To date, the majority of miRNA targets in cotton were predicted by bioinformatics approaches, and only a small portion were experimentally validated. Although no cotton cultivar is immune to Verticillium wilt, most cultivars of *G. barbadense*, such as Hai-7124, show significant advantages in Verticillium wilt resistance [17,20]. Therefore, to detect new miRNAs and their potential targets participating in the regulation of Verticillium defense responses, four sRNA libraries and two degradome libraries using RNAs from mock- and Verticillium-inoculated *Gossypium* *hirsutum* L. (*G. Hirsutum*) and *Gossypium* *barbadense* L. (*G. Barbadense*) roots were constructed and sequenced using a Solexa analyzer. A total of 140 known miRNAs and 58 novel miRNAs were identified and 107 genes sliced by 45 miRNA families were detected via degradome sequencing. The profiling of the miRNAs and their target genes provides novel information about the regulatory network of defense responses in cotton to *V. dahliae*.

## 2. Results

### 2.1. Overview of Small RNA (sRNA) Library Sequencing

**Figure 1:**
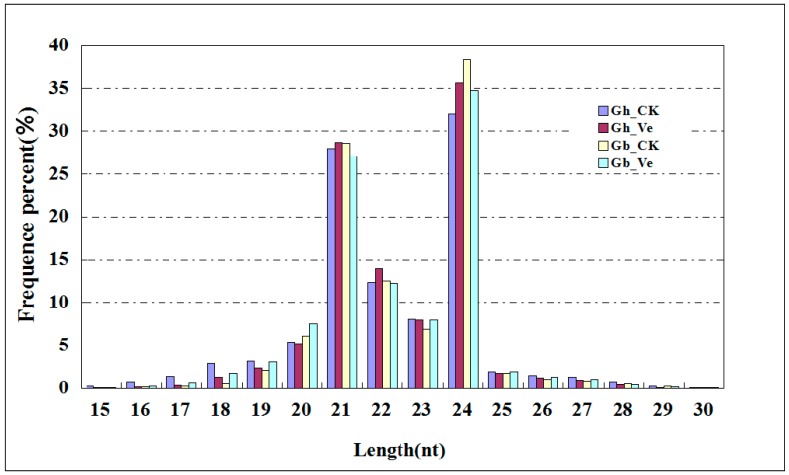
Length distribution of sRNAs in *G. hirsutum* roots and *G. barbadense* roots. Gh_CK: mock-inoculated *G. hirsutum* roots; Gh_Ve: Verticillium-inoculated *G. hirsutum* roots; Gb_CK: mock-inoculated *G. barbadense* roots; Gb_Ve: Verticillium-inoculated *G. barbadense* roots.

Four sRNA libraries were constructed and deep sequenced with total RNAs from *G. hirsutum* (Gh_CK: mock-inoculated; Gh_Ve: Verticillium-inoculated) and *G. barbadense* (Gb_CK: mock-inoculated; Gb_Ve: Verticillium-inoculated) roots. A total of about 26, 20, 21, and 20 million raw reads were obtained from Gh_CK, Gh_Ve, Gb_CK, and Gb_Ve, respectively (Table 1). After filtering out the reads of low quality and the adaptor sequences, there were approximately 26, 19, 20, and 20 million clean reads obtained in the Gh_CK, Gh_Ve, Gb_CK, and Gb_Ve libraries. To simplify the sequencing data, all identical sequence reads in each sRNA library were grouped and converted into unique sequence tags with associated counts of the individual sequence reads. There were 7,181,742; 6,033,991; 6,729,005; and 6,415,595 unique tags in the four sRNA libraries, respectively (Table 1). The length distributions of sRNAs were very similar between the four libraries (Figure 1). In these four libraries, the majority of sRNA sequences were 20–24 nt in size with 21 or 24 nt as the major size classes, which is typical for Dicer-derived products (Figure 1).

**Table 1:** Analysis of small RNA (sRNA) reads from four sRNA libraries.

Category	Library Name			
	Gh_CK	Gh_Ve	Gb_CK	Gb_Ve
Total reads	26,495,780	19,511,637	20,609,252	19,965,211
High quality	26,409,519	19,450,186	20,548,220	19,903,524
Clean reads	25,679,650	19,296,249	20,389,102	19,638,276
Unique sRNAs	7,181,742	6,033,991	6,729,005	6,415,595
Unique sRNA mapping to genome	2,235,867	1,855,605	2,078,150	1,988,015
miRNA	37,936	29,080	32,658	34,630
Unannotated	6,264,557	5,281,380	5,889,075	5,583,553

### 2.2. Identification of Known MicroRNAs (miRNAs) by sRNA Sequencing

To identify known miRNAs in the four libraries, all mappable sRNA sequences were compared with the currently known plant miRNAs in the miRBase database (release 20.0), which contains 78 known cotton miRNAs. In total, approximately 77 known cotton miRNAs belonging to 52 families were identified in the four libraries. The numbers of reads of the 77 known cotton miRNAs in the four sRNA libraries are listed in Table S1. The miRNA families miR156 and miR166 were the most abundant in the four libraries. In addition, a total of the 63 known unique miRNAs with high sequence similarity to the other known plant miRNAs, representing 50 known miRNA families, were identified in the four libraries (Table S2). These known miRNAs, with a minimal folding free energy (MFE) of the predicted hairpins ranging from −21.34 to −130.6 kcal/mol (Table S2), covered almost all the plant-conserved miRNA families. Several known but non-conserved miRNA families that have previously been identified only from one or a few plant species were also found: e.g., miR477, miR530, miR827, miR1448, miR2111, miR2947, miR2950, miR3476, and miR5083. In the present study, most of the newly identified miRNA families, such as miR168, miR403, miR477, miR828, and miR1448, were from *G. hirsutum*, while most miRNAs from *G. barbedense* had already been identified previously (Table S2).

### 2.3. Identification of Novel miRNAs in G. hirsutum and G. barbadense

To predict novel miRNAs in *G. hirsutum* and *G. barbadense*, all mappable sRNAs were BLASTed to the *G. raimondii* genome sequence and known plant miRNAs in the miRBase database (release 20.0). The sRNAs that exactly mapped to the genome sequence and unknown plant miRNAs and their flanking sequences that could be folded into a secondary structure were considered as miRNA candidates. To increase predictive accuracy, five criteria described in the experimental section were mainly used to search for novel miRNAs. In total, 58 novel miRNAs were identified in the present study (Table 2). These new miRNAs were named temporarily in the form of novel_miR_number: e.g., novel_miR_1 and their lengths were 20, 21, 22, or 23 nt (Table 2). Among these miRNAs, 34 were detected in at least two of the four sRNA libraries, and 10 were detected in all four sRNA libraries. The predicted hairpins of their precursors had a MFE ranging from −21.1 to −143.2 kcal/mol with an average of −57.5 kcal/mol. All secondary hairpin structures are listed in Table S3.

**Table 2:** Novel microRNAs (miRNAs) identified in *G. hirsutum* and *G.* *barbadense*.

miRNA	Mature Sequence	LM	Arm	LP	G + C (%)	MFE	Reads Per Million			
							Gh_CK	Gh_Ve	Gb_CK	Gb_Ve
novel_miR_1	TTGACAAGTAAAAGAACATA	20	3p	147	29.25	−23.44	0	0.98	0.74	1.07
novel_miR_2	CGAAGTCTTGGAAGAGAGTAA	21	3p	91	37.36	−39.7	49.65	0	24.82	30.3
novel_miR_3	TGGTATTGGAGTGAAGGGAGC	21	5p	70	37.14	−21.1	50.9	73.64	68.57	44.5
novel_miR_4	TGATTGAGCCGTGCCAATATC	21	3p	101	42.57	−53.9	115.81	141.22	231.64	163.25
novel_miR_5	GTGGGCGTGCCGGAGTGGTTA	21	5p	74	56.76	−26.8	110.52	96.7	34.04	36.76
novel_miR_6	CGGACTCTCAAACAGTGGAGGTA	23	5p	256	45.31	−108.08	7.63	5.44	0	12.78
novel_miR_7	CTGGACTGTCAATGGCCGGCAC	22	3p	95	45.26	−35.4	0	0	2.45	1.63
novel_miR_8	ATTAGATACTATAGCATGAGACA	23	5p	289	24.57	−46.7	3.66	0	0	9.01
novel_miR_9	CTGTCGCAGGGGAGATGGCTCGT	23	5p	141	58.16	−66.1	9.27	8.24	0	0.51
novel_miR_10	TCTAATAGAAGAATGACAAATCA	23	5p	84	20.24	−22	0	0.93	1.52	0.81
novel_miR_11	TGGGAAATGATGACAGCTTA	20	3p	193	29.53	−44.9	4.24	0	0	1.22
novel_miR_12	GCAGATGATGATAAGAATAGACA	23	5p	140	40.71	−33.3	2.26	2.23	1.77	0.66
novel_miR_13	GGGCGCCTCTTACTTGGCAGG	21	5p	132	44.7	−56.5	0.9	4.3	0	7.13
novel_miR_14	ATTAGATACTATAGCGTGAGACA	23	3p	226	37.17	−61.1	0	0	0	7.69
novel_miR_15	GCTCACTTCTCTTTCTGTCAGTT	23	3p	107	44.86	−57.7	5.33	9.74	11.87	7.84
novel_miR_16	TGCCTGGCTCCCTGAATGCCA	21	5p	104	50	−53.3	1.75	0.73	0.49	1.32
novel_miR_17	AGGTGCAGGTGCAGGCGCAGC	21	3p	146	40.41	−49.62	0	0	7.9	1.53
novel_miR_18	CGGACCCTCTAACAGTGGAGG	21	5p	214	50.47	−90.3	56.93	37.47	0	38.9
novel_miR_19	TGAGAAAGTGGAGATGGGTGG	21	3p	128	45.31	−63.9	2.18	1.71	3.87	2.6
novel_miR_20	CTCTGGTTTGACTCATTTGTA	21	5p	181	33.7	−66.8	3.12	0	0	1.63
novel_miR_21	CAAATGAGTTAGGCGAGAGGT	21	3p	170	33.53	−63.56	0	13.42	0	7.08
novel_miR_22	TCAAGCAAATCAAGGAAAGGCC	22	3p	344	38.66	−113.5	0.55	0.47	1.13	0.36
novel_miR_23	TCTGAAAAGCAATAAAGAACACA	23	3p	94	27.66	−25.7	0	0	2.55	1.78
novel_miR_24	GTGGAAGTTAGGCTGACTTAGGC	23	5p	79	45.57	−21.7	0	0	0	4.12
novel_miR_25	AGGCAGTCACCTTGGCTAAC	20	5p	180	45.56	−64.6	0	4.98	0	1.68
novel_miR_26	TTGGATGGGCGGTGTGTTTACTT	23	3p	246	28.46	−49.4	0	0	0.64	1.93
novel_miR_27	GGCAAGTTGTCCTCGGCTACA	21	3p	178	39.89	−59.96	0	1.87	0	1.12
novel_miR_28	TCCATATTTCACTATCTCTTA	21	3p	105	32.38	−48.6	11.25	16.01	72.54	22.66
novel_miR_29	TTGAACACCGAAGTAAAGCCAT	22	5p	209	41.15	−82.8	0	0	0	1.78
novel_miR_30	TGCCAAATCAGGGAAGCGAA	20	5p	148	41.22	−52.32	0	0	43.5	0
novel_miR_31	AGGCTGTGATGACGAGAAATTA	22	3p	237	30.38	−47.71	32.94	24.1	16.92	0
novel_miR_32	TTTCCAATAGAAGAATGACAAAT	23	5p	140	27.14	−54.8	1.36	1.09	1.03	0
novel_miR_33	AATCTCCCTCAAACGCTTCCAG	22	5p	118	45.76	−47.84	0	0	9.66	0
novel_miR_34	TTTGGATTGAAGGGAGCTCTA	21	3p	203	42.36	−92.45	0	0	141.2	0
novel_miR_35	TAGTGAGGATGGGAAATTTGT	21	5p	124	30.65	−49.3	34.93	0	18.78	14
novel_miR_36	GAGCTTGGAAGTGCATCCGGC	21	5p	107	45.79	−54.9	0	0	4.07	0
novel_miR_37	TCGCTTCCCTAATTTGGACGA	21	3p	148	41.22	−52.32	36.33	31.09	0	85.09
novel_miR_38	TGACTCCTAGTACAACGGCCTC	22	3p	328	32.32	−123.5	0	0	5.84	0
novel_miR_39	TTGAGCCGTGCCAATATCAATC	22	3p	108	47.22	−49.81	0	0	226.44	0
novel_miR_40	CAAAGAGTAGAGGTATTGTGC	21	5p	272	41.54	−112	0	13.89	1.23	0
novel_miR_41	ACAGGTTAGTAGAAATTAAGGTT	23	5p	115	41.74	−22.6	0	1.09	0	0
novel_miR_42	CAGAATGACCAATTTACTCTTTA	23	3p	199	24.12	−40.4	0	4.92	0	0
novel_miR_43	ACTCTCTTCCAAAGGCTTCAAG	22	5p	108	35.19	−46	0	49.91	0	0
novel_miR_44	TGGTGCAGGTCGGGAACTGAT	21	5p	139	47.48	−57.4	0	5.86	0	0
novel_miR_45	GTTCAATAAAGCTGTGGGAAG	21	3p	138	40.58	−54.6	0	100.69	0	0
novel_miR_46	CAAAAGCAATGAAGAACTGGCCA	23	5p	331	34.14	−78.9	0	1.97	0	0
novel_miR_47	ACAGTAGAAATGGATGGAATT	21	3p	153	30.07	−48	0	3.99	0	0
novel_miR_48	TTGGATGGACGGTGCATTTATCT	23	3p	295	37.29	−62.4	0	1.04	0	0
novel_miR_49	ATTATTGTTAATGTAGGAGGA	21	5p	371	31	−61.26	0	1.92	0	0
novel_miR_50	TTGTACTAGGAGTCGGATTGC	21	5p	344	33.72	−143.2	0	4.15	0	1.27
novel_miR_51	TTAGATCAAAGAGCAAACCGG	21	5p	210	36.67	−95.5	1.32	0	0	0
novel_miR_52	CGAGACTTGCGGTAGAAACAAA	22	3p	149	40.27	−35.2	6.74	0	0	0
novel_miR_53	TGGAGGCAGCGGTTCATCGATC	22	5p	110	42.73	−40.1	0	0	0	12.37
novel_miR_54	GGGACTCTCCGGACTGTTTGGTT	23	3p	148	36.49	−43.3	1.05	0	0	0
novel_miR_55	TGCCTGGCTCCCTGTATGCCA	21	5p	103	47.57	−57.4	107.56	71.93	11.92	57.64
novel_miR_56	TTTTGCCAGCTCCGCCCATTCC	22	3p	122	40.98	−45.01	66.24	87.89	0	1.73
novel_miR_57	CAGCCAAGGATGACTTGCCGG	21	5p	175	35.43	−57.4	0.97	1.66	0	0.92
novel_miR_58	TTTTTTAGTAGAAGGAGCAAAAT	23	5p	254	32.68	−56.8	0.35	0	0	0

LM: length of mature miRNA; LP: length of precursor; MFE: minimal folding free energy; Gh_CK: mock-inoculated *G. hirsutum* roots; Gh_Ve: Verticillium-inoculated *G. hirsutum* roots; Gb_CK: mock-inoculated *G. barbadense* roots; Gb_Ve: Verticillium-inoculated *G. barbadense* roots.

### 2.4. Expression Profiling of Differentially Expressed miRNAs in Response to V. dahliae Infection

**Figure 2:**
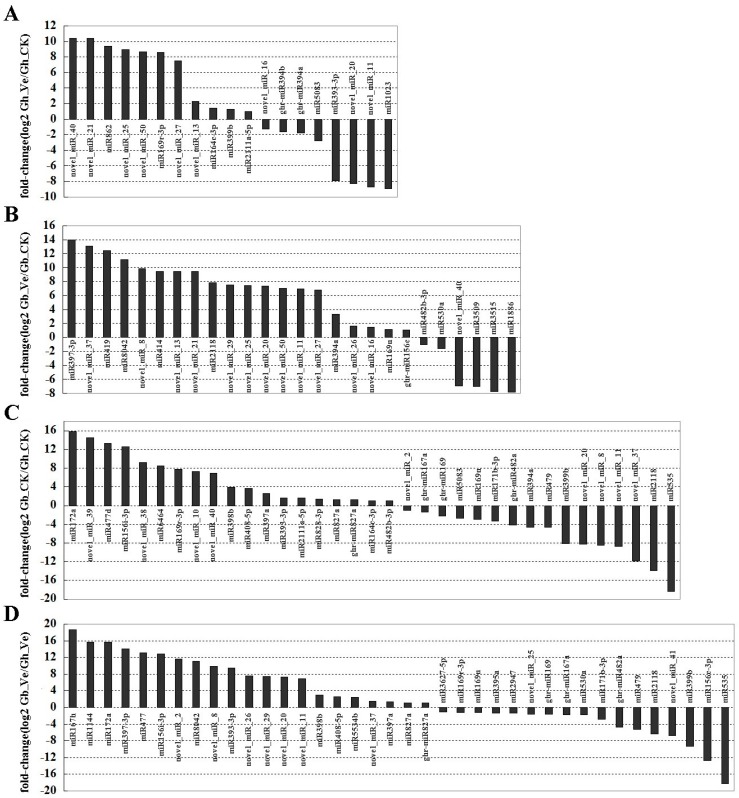
Expression profiles of differentially expressed miRNAs by high-throughput sequencing. (**A**) Differentially expressed miRNAs between Gh_Ve and Gh_CK libraries; (**B**) Differentially expressed miRNAs between Gb_Ve and Gb_CK libraries; (**C**) Differentially expressed miRNAs between Gb_CK and Gh_CK libraries; (**D**) Differentially expressed miRNAs between Gb_Ve and Gh_Ve libraries. Gh_CK: mock-inoculated *G. hirsutum* roots; Gh_Ve: Verticillium-inoculated *G. hirsutum* roots; Gb_CK: mock-inoculated *G. barbadense* roots; Gb_Ve: Verticillium-inoculated *G. barbadense* roots.

The expression profiles of miRNAs were analyzed and compared between the four libraries based on the number of clean reads generated from the high-throughput sequencing. After normalization, the reads of the tags of each miRNA family as “reads per million”, *p*-value < 0.01, and the absolute value of |log2^Ratio^| ≥ 1 were considered to indicate the statistical significance of miRNA expression. Interestingly, many miRNAs were differentially expressed between the libraries. miRNA expression between the mock- and Verticillium-inoculated treatments for the two cotton species was first analyzed. In *G. hirsutum* roots, a total of 19 miRNAs representing nine novel miRNAs were identified as *V. dahliae*-responsive miRNAs (Figure 2A). Among them, 11 miRNAs were preferentially expressed in the Gh_Ve treatment and eight were preferentially expressed in the Gh_CK treatment. In *G. barbadense* roots, a total of 26 miRNAs representing 13 novel miRNAs were identified as *V. dahliae*-responsive miRNAs (Figure 2B). Among them, 20 were of higher abundance and only six were of lower abundance in the Gb_Ve treatment. Notably, novel_miR_13, novel_miR_21, novel_miR_25, novel_miR_27, and novel_miR_50 were of higher abundance in the roots of both cotton species after 24 h of treatment.

The miRNA expressions between the two cotton species with mock- and Verticillium-inoculated treatments were also analyzed. We made a comparative analysis of miRNA expression between the two cotton species with mock-inoculated treatment (Figure 2C). It was shown that a total of 35 miRNAs had a species-specific expression. Among them, 16 miRNAs were preferentially expressed in *G. hirsutum* roots and 19 were preferentially expressed in *G. barbadense* roots. Because of the genotype-specific expression of miRNAs under mock-inoculated treatment, it was plausible to assume that some miRNAs had preferential expression in one of the two species when both of them were inoculated by Verticillium. Thus, we compared the expression levels of miRNAs between Gb_Ve and Gh_Ve libraries. We found that 38 miRNAs had species-specific expression in Verticillium-inoculated treatments (Figure 2D). Among them, 17 miRNAs were preferentially expressed in *G. hirsutum* roots and 21 were preferentially expressed in *G. barbadense* roots. Interestingly, most of the species-specific-expressed novel miRNAs (e.g., novel_miR_2, novel_miR_8, novel_miR_11, novel_miR_20, novel_miR_26, novel_miR_29, and novel_miR_37) were significantly preferentially expressed in Verticillium-inoculated *G. hirsutum* roots.

**Figure 3:**
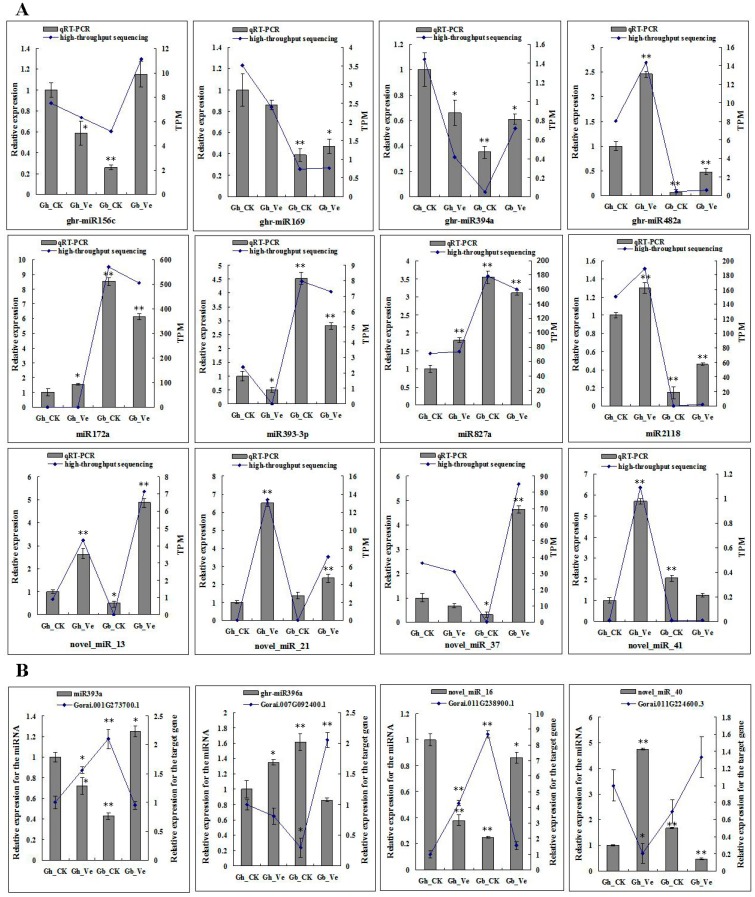
Relative expression analysis of miRNAs and target genes in the *G. hirsutum* and *G. barbadense* roots. (**A**) Relative expression analysis of miRNAs by qRT-PCR analysis and high-throughput sequencing; (**B**) Relative expression analysis of miRNAs and their target genes by qRT-PCR analysis. Gh_CK: mock-inoculated *G. hirsutum* roots; Gh_Ve: Verticillium-inoculated *G. hirsutum* roots; Gb_CK: mock-inoculated *G. barbadense* roots; Gb_Ve: Verticillium-inoculated *G. barbadense* roots; TPM: Tags per million clean reads. U6 snRNA and *Ubiquitin1* were chosen as endogenous control genes. Error bars indicate standard deviation of three biological replicates.* and ** indicate significant differences relative to the Gh_CK at *p* < 0.05 and *p* < 0.001 by Student’s *t*-test, respectively.

To validate the existence and expression patterns of the predicted miRNAs in mock- and Verticillium-inoculated cotton roots, four novel miRNAs, as well as eight representative known miRNAs, were selected for qRT-PCR analysis. Although some non-conserved and novel miRNAs were identified in low read number or were undetectable in one or two sRNA libraries by Solexa sequencing, the 12 selected miRNAs were detected by qRT-PCR. The qRT-PCR results of those miRNAs were quite consistent with the results from the sequencing data, and confirmed the changes in miRNA expression in response to *V. dahliae* infection (Figure 3A). Furthermore, to confirm the causality of the miRNA expression patterns and their target gene, we studied the expression of four miRNAs and their target genes by qRT-PCR in *G. hirsutum* and *G. barbadense* roots. Among the target mRNAs tested, four targets showed a significant inverse correlation in expression with their corresponding miRNAs (Figure 3B).

### 2.5. Target Genes of miRNAs Identified by Degradome Analysis

**Figure 4:**
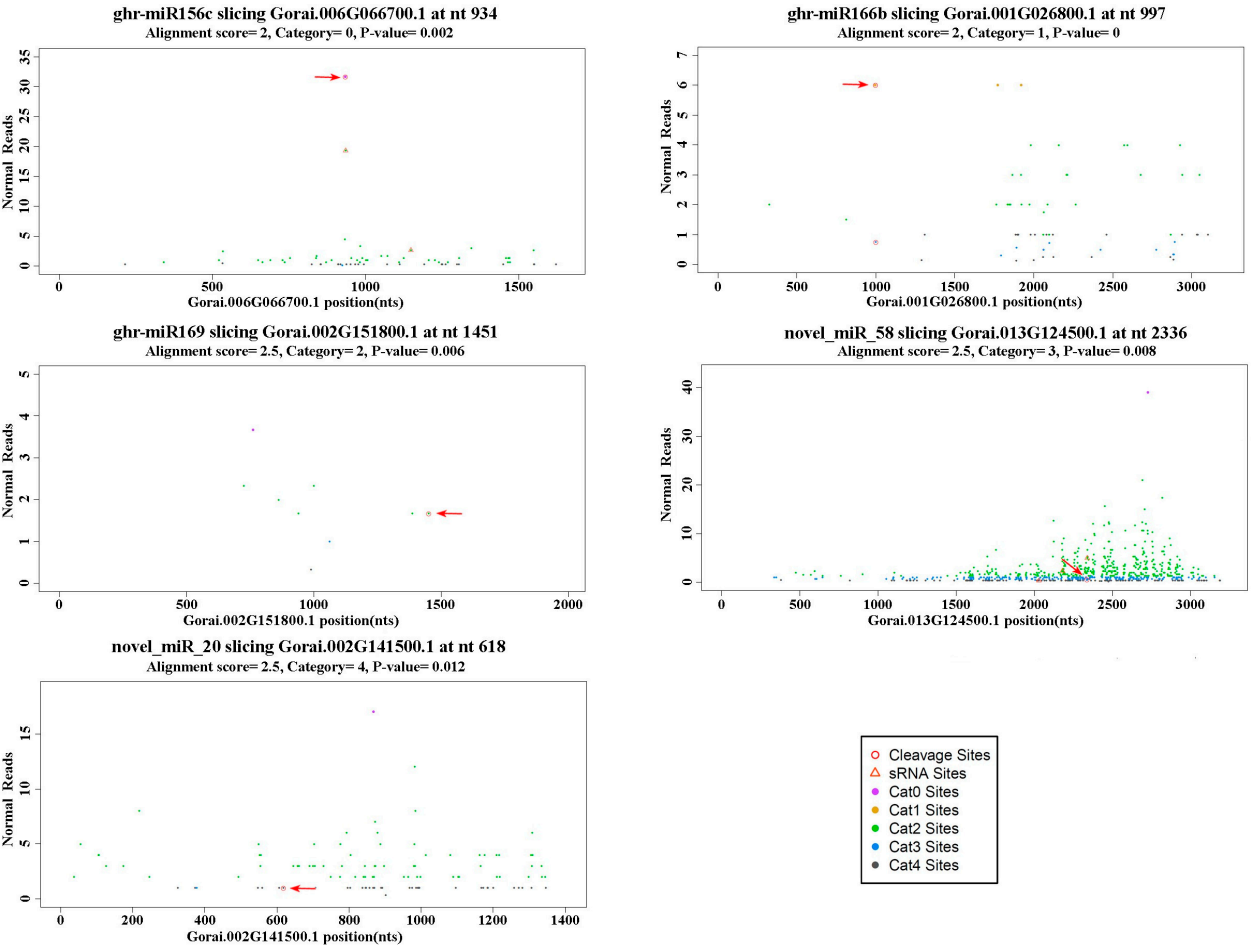
Target plots (t-plots) of the representative miRNA targets in different categories confirmed by using degradome sequencing. Normal Reads: repeat normalized number of 5′ends at that position for target gene. Red arrows indicate the target genes cleavage sites.

In the present study, degradome sequencing was used to search for the target genes of identified miRNAs in cotton. According to the relative abundance of tags at the predicted miRNA target sites, the identified targets were grouped into five categories (0–4) as described by Yang [21]. Category “0” was defined as >1 raw read at the position, with abundance at the position equal to the maximum on the transcript, and with only one maximum on the transcript. Category “1” was defined as >1 raw read at the position, with abundance at the position equal to the maximum on the transcript, and with more than one maximum position on the transcript. Category “2” included >1 raw read at the position, and abundance at the position less than the maximum but higher than the median for the transcript. Category “3” comprised transcripts with >1 raw read at the position, and abundance at the position equal to or less than the median for the transcript. Category “4” showed only one raw read at the position [21]. The representative miRNAs and corresponding targets are shown in Figure 4, in which the red arrows indicate the cleavage sites. A total of 83 and 24 targets were predicted to be cleaved by 31 known and 14 novel miRNA families, respectively (Table 3 and Table S4). Among the identified targets of known miRNAs, squamosa-promoter binding protein (SBP) and squamosa-promoter binding protein-like (SPL) transcription factor genes, NAM, ATAF, and CUC (NAC) domain-containing protein genes, Class III homeodomain leucine-zipper (HD-Zip) protein genes, APETALA2 (AP2)-like factor genes, F-box/RNI-like superfamily protein (TIR1) genes, and growth-regulating factor (GRF) genes were targeted by several conserved miRNA families (including miR156, miR164, miR166, miR172, miR393, and miR396) that play significant roles in gene regulation (Table S4). ghr-miR7502, ghr-miR7505, ghr-miR7509, ghr-miR7510a, and ghr-miR7514, which were only reported in *G. hirsutum*, were predicted to cleave Nudix hydrolase, ATP binding and tetratricopeptide repeat-like superfamily protein, glutamate receptor 2 isoform 1, peroxidase superfamily protein, and RHO guanyl-nucleotide exchange factor 7 (Table S4). Among the identified targets of novel miRNAs, ubiquitin carboxyl-terminal hydrolase family protein gene was targeted by novel_miR_12; auxin response factor genes were targeted by novel_miR_16; purine permease 3 and ferrochelatase 2 genes were targeted by novel_miR_20; and TT12-2 (a T-DNA insertion line) MATE (multidrug and toxic efflux) transporter gene was targeted by novel_miR_24 (Table 3). Interestingly, we also found that leucine-rich repeat (LRR) containing protein genes and LRR and NB-ARC (nucleotide-binding adaptor shared by APAF-1, R proteins, and CED-4) domains-containing disease resistance-like protein genes that play important roles in plant defense against pathogens were targeted by novel_miR_28 and novel_miR_56, respectively (Table 3). Unfortunately, target genes of some known miRNAs and novel miRNAs identified through deep sequencing were not detected in the present degradome analysis.

**Table 3:** Target genes of novel miRNAs identified by degradome sequencing in cotton.

miRNA	Target Gene	Cleavage Site	Categoty	Norm Reads		Target Description
				Gb	Gh	
novel_miR_12	Gorai.005G020900.1	1117	1	0	3	Ubiquitin carboxyl-terminal hydrolase family protein
novel_miR_16	Gorai.003G142500.1	1315	0	58	54	Auxin response factor 10 isoform 1
	Gorai.013G267100.1	1416	0	60	31	Auxin response factor 10 isoform 1
	Gorai.010G046000.1	1638	0	16	19	Auxin response factor 17
	Gorai.006G166400.1	1773	0	36	12	Auxin response factor 19
	Gorai.011G238900.1	1502	0	316	557	Auxin response factor 19
	Gorai.012G004800.1	1679	0	110	160	Auxin response factor 19
novel_miR_20	Gorai.002G141500.1	618	4	1	0	Purine permease 3
	Gorai.003G066400.1	1545	4	1	0	Ferrochelatase 2 isoform 1
	Gorai.008G199200.1	1792	4	1	0	Uncharacterized protein TCM_014264
novel_miR_21	Gorai.009G146400.1	1251	0	7	0	Uncharacterized protein TCM_029129
novel_miR_24	Gorai.006G008600.5	496	0	1	0	TT12-2 MATE transporter
novel_miR_26	Gorai.007G279100.4	319	2	0	2	Intracellular protein transport protein USO1
	Gorai.012G074700.1	79	2	0	2	Serine hydroxymethyltransferase 6 isoform 3
novel_miR_28	Gorai.003G163700.1	1250	0	13	0	Leucine-rich repeat containing protein
novel_miR_29	Gorai.008G010200.1	855	2	7	5	Glutathione S-transferase 7 isoform 1
	Gorai.008G120500.1	1587	3	0	2	Fringe-related protein, putative
novel_miR_30	Gorai.005G163400.1	1744	0	28	0	Uncharacterized protein TCM_010813
novel_miR_40	Gorai.011G224600.3	1555	1	0	1	Inositol 1,3,4-trisphosphate 5/6-kinase 4
novel_miR_48	Gorai.007G279100.4	319	2	0	2	Intracellular protein transport protein USO1
	Gorai.012G074700.1	79	2	0	2	Serine hydroxymethyltransferase 6 isoform 3
novel_miR_56	Gorai.007G319800.1	613	0	10	1	LRR and NB-ARC domains-containing disease resistance-like protein
novel_miR_57	Gorai.004G172100.1	1219	0	19	26	Nuclear transcription factor Y subunit A-1, putative isoform 1
novel_miR_58	Gorai.013G124500.1	2336	3	1	1	Bacterial-induced lipoxygenase

Normal Reads: repeat normalized number of 5′ends at that position for target gene.

## 3. Discussion

*Verticillium dahliae* Kleb. is a soil-borne fungal pathogen that causes vascular wilt of more than 200 dicotyledonous plant species, including cotton crops [14]. Sea-island cotton (*G. barbadense* L.) exhibits relative resistance to Verticillium wilt, but upland cotton (*G. hirsutum* L.), the main species cultivated on a large scale, is sensitive to this disease [17,22]. Previous study has shown that a number of miRNAs and other small non-coding RNAs were involved in response to *V. dahliae* infection in *G. hirsutum* and *G. barbadense* roots [23]. However, most of these miRNA targets were previously predicted in cotton [23], and only a few miRNA targets have been identified experimentally [23,24]. The functions of most of these miRNAs in relation to the regulation of Verticillium defense responses remain unknown and further studies must be conducted. In the present study, the two cotton species *G. hirsutum* and *G. barbadense* were used as models to study the miRNA functions associated with the regulation of Verticillium defense responses. We constructed and sequenced four sRNA libraries and two degradome libraries from mock- and Verticillium-inoculated cotton roots. In total, 140 known miRNAs and 58 novel miRNAs were detected in *G. hirsutum* and *G. barbadense* by deep sequencing. In addition, 107 target genes of 45 miRNA families were identified by degradome library sequencing. The present study is the first to report comprehensive identification of miRNAs and their targets involved in cotton response to *V. dahliae* by using high-throughput sequencing and degradome analysis. This will provide useful information for improving the Verticillium wilt resistance of economically important crops.

miRNAs have been identified experimentally in many plant species, especially in model plants. In plants, some miRNAs seem to be universally expressed, while others are present in only a few species. According to previous reports, conserved miRNAs are present throughout at least one major ancient clade of land plants, and non-conserved miRNAs, with a limited phylogenetic distribution, are characterized by primarily being single-copy genes [25]. In this study, most known miRNAs were conserved in other species and had been previously predicted. Many other studies have shown that a number of the most conserved miRNA targets common among the data sets include many transcription factors: SBP, SPL, auxin response factors (ARF), MYB (Myeloblastosis), NAC, TCP (Teosinte-like 1, Cycloidea, and Proliferating cell factor 1), NF-Y (Nuclear Factor Y), GRF, HD-ZIP, PPR (Pentatricopeptide Repeat), and AP2-like factors. It is possible that conserved miRNAs play a crucial role in universal mechanisms of regulation in different plant species and may help us understand the evolutionary relationships between cotton and other plants. Some known but non-conserved miRNAs (e.g., miR477, miR530, miR827, miR1448, miR2111, miR2947, miR2950, miR3476, and miR5083), which were also detected in the present study, have been only identified in one or few plant species so far. In addition, 58 novel miRNAs with a lower abundance than that of conserved miRNAs were identified by using universal rules for miRNA annotation [26]. They are likely to be cotton-specific miRNAs, which are classified into non-conserved miRNAs. It seems likely that these miRNAs evolved relatively recently [5], and may function only to regulate gene expression during Malvaceae- or cotton-specific biological processes.

miRNAs regulate gene expression at the transcriptional and post-transcriptional levels via mRNA cleavage or translational repression. In higher plants, miRNAs mediate gene silencing mainly by slicing mRNAs [27]. miRNA-directed cleavage leaves a 5ʹ-uncapped 3ʹ-fraction of the sliced genes. Therefore, the powerful tool of degradome sequencing was applied to identify miRNA target genes in many species with greater throughput [28,29,30]. In our study, this experimental approach was performed to identify target genes for known and novel miRNAs in cotton. As expected, a number of target genes were predicted to be cleaved by known and novel miRNA families. Many of the identified target genes of known conserved miRNAs belong to diverse gene families of transcription factors (e.g., MYB, NAC, HD-ZIP and AP2-like factor) which are known to regulate diverse aspects of plant growth and development as well as the response to *V. dahliae* infection [31,32]. In addition, we found some novel miRNA targets (e.g., ubiquitin carboxyl-terminal hydrolase family protein, purine permease 3, TT12-2 MATE transporter, intracellular protein transport protein USO1, glutathione S-transferase 7, and bacterial-induced lipoxygenase), suggesting a new feature of miRNA regulation in cotton. The novel miRNAs and their targets might offer useful information in potential future studies on how miRNAs and their targets are involved in the response to *V. dahliae* infection, which should be further investigated. However, the target genes for some known miRNAs and more novel miRNAs were not detected in the present degradome analysis. It is possible that the levels of these sliced targets were too low to detect, or some miRNAs might inhibit target gene expression through translational repression [33,34]. In summary, degradome analysis has greatly accelerated the identification of miRNA targets and has sped up research on miRNA–target interactions.

In our study, a number of the miRNAs exhibited altered expression in *G. hirsutum* and *G. barbadense* roots after infection with *V. dahliae*, indicating that *V. dahliae* infection could disrupt global gene regulatory networks during cotton development. These *V. dahliae*–responsive miRNAs might contribute to species-specific regulation, act as “early” regulators of signal transduction or be advantageous for adaptation to stressed environments [23,35]. In *Populus*, Ptc-miR482 was validated to cleave disease resistance protein genes, which were involved in the resistance of plants to biotic and abiotic stresses [36,37]. Previous studies have shown that the NBS (nucleotide-binding site)-LRR resistance gene might contribute to *V. dahliae* resistance in cotton [31,38]. In this study, ghr-miR482a, which targets LRR and NB-ARC domain-containing disease resistance–like proteins, was expressed at a very low level in *G. barbadense* mock- and Verticillium-inoculated roots compared with *G. hirsutum* roots, suggesting its possible role in relative resistance to Verticillium wilt of *G. barbadense*. The expression profiles of miR1886, miR3509, and miR3515 were down-regulated, while miR419 and miR2118 were up-regulated in *G. barbadense* roots after infection with *V. dahliae*. This was consistent with a previous study that showed that many miRNAs had a species-specific expression after infection of the fungal pathogen Verticillium in *G. hirsutum* and *G. barbadense* [23]. In plants, ARFs were involved in regulating the auxin signaling pathway, which plays an important role in growth, development, and environmental responses. In *Arabidopsis*, repression of auxin signaling could restrict *P. syringae* growth, implicating auxin in disease susceptibility and miRNA-mediated suppression of auxin signaling in disease resistance [8,39]. novel_miR_16, which targets ARF10, ARF17, and ARF19, was also induced by *V. dahliae*, indicating that it also played an important role in plant disease resistance, but this requires further experimental confirmation. There are still many other miRNAs involved in Verticillium-infection response; however, their target genes were not detected in the present degradome analysis and their functions in plants are unknown. Future analysis of target genes and molecular components downstream could help us to understand the significance of their roles in the process.

## 4. Experimental Section

### 4.1. Plant Material and Total RNA Isolation

The *G. barbadense* L. variety Hai-7124 (resistant) and *G. hirsutum* L. variety Yi-11 (susceptible) seeds were grown in pasteurized sand which was placed in a greenhouse (day temperature 28 °C, night temperature 25 °C, and relative humidity 60%) with a photoperiod of 14/10 h of light/dark and watered with Hoagland culture liquid every 3 day.

*Verticillium dahliae* isolates were provided by the College of Plant Protection, Shan Dong Agricultural University. After in-plate activation, *V. dahliae* was transferred to Czapek Broth liquid medium and cultured for 15 day (200 rpm, 25 °C). Then, after filtration through four layers of sterile gauze, *V. dahliae* was diluted to approximately 10^7^ spores per ml of suspension with sterile water before inoculation.

The cotton seedlings with two fully expanded leaves were inoculated with *V. dahliae* by root dip inoculation into a suspension of fungal spores for 5 min and were then returned to their original pots. Control plants were not inoculated but were otherwise treated and sampled with distilled water in the same way. After 24 h of inoculation, the roots of both pathogen-infected and control seedlings were harvested immediately, frozen in liquid nitrogen, and stored at −80 °C for RNA isolation. In each case, samples were harvested and pooled from 20 individual plants. Total RNA was isolated from each sample using a modified CTAB (cetyltrimethylammonium bromide) method [40].

### 4.2. sRNA Library and Degradome Library Construction and Sequencing

sRNA library construction and deep sequencing were performed as described by Hafner [41]. A 20 µg aliquot of total RNA was sent to the Beijing Genomics Institute (Shenzhen, China) where the libraries were constructed and sequenced using an Illumina Genome Analyzer (Illumina, San Diego, CA, USA). Briefly, the sRNAs (~18–30 nt) were purified from 10 µg of total RNA by polyacrylamide gel electrophoresis, and ligated first to a 5ʹ-RNA adaptor and then to a 3ʹ-RNA adaptor. A reverse transcription reaction was followed by several cycles of PCR to obtain sufficient product for Sequencing by Synthesis (SBS) sequencing via Solexa technology.

Two cotton degradome libraries (Gh and Gb) were constructed based on a method previously described [28]. Briefly, total RNAs were respectively extracted from Gh_CK and Gh_Ve, and mixed at an equal molar ratio as one sample for Gh degradome library construction. Total RNAs from Gb_CK and Gb_Ve were mixed for Gb degradome library construction in the same way. Approximately 200 µg of the mixed total RNA was used for polyadenylation using the Oligotex mRNA kit (Qiagen, Valencia, CA, USA), and then a 5ʹ-RNA oligonucleotide adaptor containing an MmeI recognition site was ligated to the 5ʹ-phosphate of the poly(A+) RNA by T4 RNA ligase. This was followed by purification of the ligated products using the Oligotex kit. Subsequently, five PCR cycles were performed on the products of a reverse transcription reaction which were then digested with MmeI and ligated to a 3ʹ-double DNA adaptor. Finally, the ligation products were amplified with 20 PCR cycles, gel-purified and subjected to SBS sequencing by the Illumina Genome Analyzer.

### 4.3. Analysis of Sequencing Data

Bioinformatic analysis of sRNA and degradome sequencing data was based on a method described previously [42]. For the sRNA sequencing data, the unique RNA sequences that perfectly matched the cotton genome were subjected to subsequent analysis. RNA reads showing sequences identical to known miRNAs from the miRBase 20.0 database were picked up as the miRNA dataset of cotton. Sequences matching non-coding rRNA, tRNA, snRNA, and snoRNA in the Rfam database were removed. Reads overlapping with exons of protein-coding genes were excluded to avoid mRNA contamination. The remaining sequences were used to predict their secondary structures by using the mfold web server [43,44]. A potential miRNA precursor must meet certain criteria: (1) Both a candidate miRNA and its corresponding reverse sequence (miRNA*) must be detected in the present high-throughput sequencing; (2) The candidate miRNA and miRNA* sequences must be found on the stem, and the number of mismatched bases between them must be less than four; (3) Within the miRNA/miRNA* duplex, the number of asymmetric bulges must be one or fewer, and the number of bases in the asymmetric bulges must fewer than two; (4) The miRNA and miRNA* should be located in opposite stem-arms and form a duplex with two nucleotide 3′overhangs; (5) The potential miRNA precursor must have higher negative minimal folding energy (MFE) with the MFE <−18 kcal/mol.

To investigate the differentially expressed miRNAs between libraries, each identified miRNA read count was normalized to the total number of miRNA reads in each given sample and multiplied by a million. Then, the Bayesian method was applied to infer the statistical significance value [45]. After the Bayesian test, if the *p*-value <0.01 and the absolute value of |log2^Ratio^| ≥ 1, a specific miRNA was considered to be differentially expressed.

For the degradome sequencing data, 20–21 nt sequences of high quality were collected for subsequent analysis. The unique reads that perfectly matched cotton cDNA sequences were retained. The 15-nt of sequence upstream and downstream of the 5ʹ-end of matched reads was extracted to constitute 30-nt sequence tags for searching corresponding miRNA. The CleaveLand pipeline [27] was used to align the 30-nt sequence to cotton-known miRNAs from miRBase20.0 database and our newly identified miRNAs. All alignments with scores up to seven and no mismatches at the cleavage site (between nucleotides 10 and 11) were considered candidate targets.

### 4.4. qRT-PCR

To validate the presence and expression of the identified miRNAs and target genes, 16 miRNAs and four target genes were selected for qRT-PCR analysis. The expression profile of miRNAs and target genes were assayed in pathogen- and mock-infected roots of Hai-7124 and Yi-11 by SYBR^®^ Premix Ex TaqTM II (TAKARA, Dalian, China) on Bio-RAD iCycler iQ5 Machine. The primers used for qRT-PCR are listed in Table S5. qRT-PCR were performed using the One Step PrimeScript^®^ miRNA cDNA Synthesis Kit (TAKARA) and using 12.5 µL of SYBR^®^Premix Ex TaqTM II (2×), 1 µL of PCR forward primer (10 µM), 1 µL of PCR reverse primer (10 µM), and 2 μL of fivefold diluted cDNA template in a 25-µL system with the following cycling profile: 95 °C at 30 s, followed by 40 cycles of 95 °C at 15 s and 60 °C at 30 s. All reactions of qRT-PCR were repeated three times for each sample. U6 snRNA and *Ubiquitin1* gene were used as the internal control genes. All the gene expression data were obtained from three individual biological replicates and processed according to strict statistical methods [46]. Statistical significance was evaluated using a Student’s *t*-test analysis.

## 5. Conclusions

In this study, four sRNA libraries and two degradome libraries were constructed from *G. hirsutum* and *G. barbadense* roots with and without *V. dahliae* infection for deep sequencing. A large number of miRNAs were identified in both species, including 58 novel and 140 known miRNAs. Meanwhile, 107 genes sliced by 45 miRNA families were detected via degradome sequencing. The differential patterns of miRNAs expression are a valuable resource for further studies on post-transcriptional gene regulation in the defense response of cotton to Verticillium wilt. Thus, the present study might provide valuable clues for exploring miRNA-mediated regulatory networks in Verticillium defense response.

## Supplementary Materials

Supplementary materials can be found at http://www.mdpi.com/1422-0067/16/07/14749/s1.
